# A Review of Microfluidic Devices for Rheological Characterisation

**DOI:** 10.3390/mi13020167

**Published:** 2022-01-22

**Authors:** Francesco Del Giudice

**Affiliations:** Department of Chemical Engineering, Faculty of Science and Engineering, School of Engineering and Applied Sciences, Swansea University, Swansea SA1 8EN, UK; francesco.delgiudice@swansea.ac.uk

**Keywords:** rheometry, viscoelasticity, microfluidics

## Abstract

The rheological characterisation of liquids finds application in several fields ranging from industrial production to the medical practice. Conventional rheometers are the gold standard for the rheological characterisation; however, they are affected by several limitations, including high costs, large volumes required and difficult integration to other systems. By contrast, microfluidic devices emerged as inexpensive platforms, requiring a little sample to operate and fashioning a very easy integration into other systems. Such advantages have prompted the development of microfluidic devices to measure rheological properties such as viscosity and longest relaxation time, using a finger-prick of volumes. This review highlights some of the microfluidic platforms introduced so far, describing their advantages and limitations, while also offering some prospective for future works.

## 1. Introduction

Rheology is defined as the science of deformation and flow [1], and it often features the study of liquid properties under an applied external flow. The rheology of liquids has become critical for the optimisation of operating conditions and equipment design across a variety of industrial processes, while also finding application in the regulation of our body functions. For instance, the consistency of a certain toothpaste or detergent brand in respect to another one determine our inclination in using either one or the other. In a similar fashion, the flow of liquids inside our body, such as blood or urine, provides an indication of our overall health. The fact that liquids are ubiquitous in our daily life has prompted a significant development of technological platforms, called rheometers, for the measurement of rheological properties.

There are several types of rheometers, depending on both the imposed flow and the working principle [1,2]. For instance, when interested in understanding the flow of complex fluids in pipelines (a very important industrial process), the properties of liquids under shear flow are essential to optimise the pipe design and to select the appropriate pump. Similarly, in the case of extrusion processes, the properties under extensional flow may be more relevant than the shear ones. For this reason, both shear and extensional rheometers have been introduced; for each category, several types of rheometers have also been developed, including the rotational rheometer, the capillary rheometer, the capillary breakup extensional rheometer, and so on [2]. Despite their appealing for the rheological characterisation of liquids, rheometers are affected by some limitations. Rheometers are generally bulky and cannot be often considered ‘easy-to-use’ instruments, as they require significant training to operate them, as well as requiring significant knowledge to properly perform the measurements and to analyse the resulting data, discriminating between valid and invalid data [1,3]. In many cases, volumes of several milliliters are required to perform a measurement, thus being a hindrance for the study of precious or expensive materials. Whenever little sample can be used in conventional rheometry, for instance when employing rotational geometries with diameters of 25 mm and below, the sensitivity of the rheometer to small torque values decreases significantly [3]. In practical terms, this may mean that viscosity values at low imposed shear-rates cannot be evaluated, or that properties such as the longest relaxation time cannot be measured. In addition, rheometers cannot be easily integrated with other technologies, thus not being very flexible.

The advent of microfluidics has made a significant impact across several applications spanning different fields [4]. Microfluidic devices require a little sample to perform a measurement, and they could be easily integrated with other systems, while also allowing a straightforward optical access via conventional optical microscopes, thanks to the soft lithography fabrication technique [5]. It did not take long before microfluidic devices for rheological characterisation were introduced with the specific purpose of addressing the limitations of conventional rheometry. Hereafter, microfluidic devices employed for the rheological characterisation of liquids are called microfluidic rheometers.

The goal of this review is to provide an overview of the microfluidic rheometers developed over the years, together with their advantages and limitations. In fact, several microfluidic devices have been introduced to measure rheological properties such as shear-viscosity, longest relaxation time, first normal stress difference and extensional viscosity, for several dilute polymer solutions and for several biofluids, proving their advantages over conventional rheometers. This review does not describe microfluidic devices where complex fluids have been used for a diverse set of applications but rather microfluidic devices for the measurement of the rheological properties of complex fluids. The review is structured as it follows: Section 2 summarises some basic concepts of rheology. In Section 3, microfluidic rheometers in shear flow are reviewed and are divided according to their working principle. Section 4 focuses on microfluidic rheometers in extensional flow, while Section 5 provides an overview of microfluidic devices integrated to other techniques. Finally, some conclusions and perspective are presented.

## 2. Essential Concepts in Rheology

Before reviewing the advancements in microfluidic devices for rheological applications, some essential concepts of rheology, which may not be familiar to the reader, are briefly discussed. The interested reader can look at the detailed rheology book edited by Macosko [2] or the one edited by Barnes [1], for further details.

**Figure 1:**
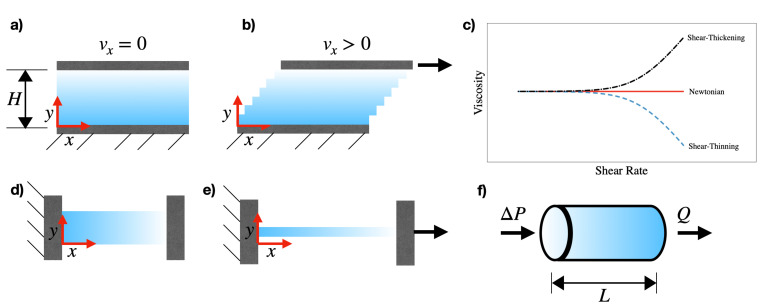
(**a**) Fluid between two parallel plates in stationary conditions. (**b**) A force is applied to the upper plate, leading to a shear flow along the *y*-direction. (**c**) Examples of Newtonian liquids with viscosity independent of the shear rate (solid red line), shear-thinning liquids having viscosity that decreases when increasing the shear rate (dashed blue line) and shear-thickening liquids having viscosity that increases when increasing the shear rate (dot-dashed black line). (**d**) Fluid between two parallel plates arranged horizontally in stationary conditions. (**e**) A force is applied to the plate on the left, thus leading to an extensional flow along the *x*-direction. (**f**) Schematic of the flow in a cylindrical channel, encountered in capillary rheometry.

Let us consider the case of a fluid confined between two stationary parallel plates with area *A*, having a gap *H*, such that $H<<\sqrt{A}$ (Figure 1a). When a force is applied to the upper plate, it moves along the *x*-direction. Because of the no-slip conditions on both the stationary and the moving plate, adjacent layers of fluids move with different velocities, leading to the so-called *shear* flow (Figure 1b). From this simple experiment, we define the shear stress τ=F/A and the shear rate as $\dot{\gamma }=v_{x}/H$. The shear viscosity η is then defined as:

(1) $$\eta =\frac{\tau }{\dot{\gamma }}.$$

For Newtonian fluids, the viscosity is constant with the shear rate (red solid line in Figure 1c). Non-Newtonian fluids, instead, display a shear viscosity that changes with the shear rate: for instance, shear-thinning liquids present a shear viscosity that decreases when increasing $\dot{\gamma }$ (blue dashed line in Figure 1c), while shear-thickening liquids present a viscosity that increases when increasing $\dot{\gamma }$ (black dot-dashed line in Figure 1c). There is also the special case of fluids having a constant shear viscosity over a wide range of shear rate values, while also displaying elastic properties: these fluids are non-Newtonian and are often referred as ‘second order’ [2] or simply ‘Boger’ [6] liquids.

Let us now consider the case of two stationary plates with area *A* arranged along an horizontal line, with liquid in between them (Figure 1d). By applying a normal force $F_{N}$ to the plate on the right, the liquid elongates along the *x*-direction, while shrinking along the *y*-direction because of the continuity equation [7] (Figure 1e): this type of flow is called *elongational* or *extensional*. By defining the normal stress as $\tau =F_{N}/A$ and the strain rate as $\dot{\epsilon }=dv_{x}/dx$, we can define the elongational viscosity $\eta_{el}$ as:

(2) $$\eta_{el}=\frac{\tau_{N}}{\dot{\epsilon }}.$$

Shear and extensional viscosity values are generally different, because they are the result of entirely different flow conditions. For Newtonian liquids, the ratio between $\eta_{el}$ and η was found to be constant and equal to $\eta_{el}/\eta =3$ [1,2], which is also called Trouton ratio. For non-Newtonian liquids, instead, the Trouton ratio is preserved only when the fluid is subjected to small deformations (also called, linear regime [1,2]). At large deformations, instead, there are significant departures from the Trouton ratio, with the elastic viscosity being significantly larger than the Newtonian one.

The elasticity of complex solutions in shear flow can be assessed by performing small angle oscillatory shear (SAOS) experiments [1,2]. In this case, the fluid is loaded between two parallel plates, with the bottom one stationary and the upper one oscillating at a certain angular frequency ω in order to impose a small deformation γ to the sample. As a consequence of this flow, two parameters called the storage modulus $G^{\prime }$ and the loss modulus $G^{\prime \prime }$ are measured: the first one provides a quantitative measurement of the elastic component, while the second one provides the viscous component of the fluid (similarly to the shear-viscosity). A characteristic parameter that can be used to describe the elasticity of the complex fluid is the *longest relaxation time* λ [1,2]. This parameter can be directly estimated from the oscillatory shear experiments in the following way [1,2]. According to the well-established relations in polymer physics [8], the storage modulus $G^{\prime }$ and the loss modulus $G^{\prime \prime }$ scale with slopes 1 and 2, respectively, with the angular frequency ω in a log–log scale, for small values of ω (the so-called, terminal region). The intersection between these two slopes provides an estimate of λ [1,2]. It is worth mentioning that, very often, the relaxation time is estimated as the point when $G^{\prime }$ and $G^{\prime \prime }$ cross-over; however, this value does not always correspond to the longest relaxation time but just to another relaxation time, as complex fluids tend to display a spectrum of relaxation time values [8]. The value of λ is very important to characterise the elasticity of complex fluids; however, it is often very difficult to measure for unentangled polymer solutions, because of the intrinsic limitations of conventional rheometers [3]. Another important parameter for the evaluation of the complex fluid elasticity is the first normal stress difference $N_{1}$, defined as [1,2]:

(3) $$N_{1}=\tau_{xx}-\tau_{yy},$$

where $\tau_{xx}$ is the normal component of the stress tensor along the flow direction, *x*, and $\tau_{yy}$ is the normal component of the stress tensor along the shear direction, *y*. Assuming that the fluid is subjected to small deformations, it is possible to write [1,2]:

(4) $$N_{1}=\lambda {\dot{\gamma }}^{2}.$$

Similarly, it is possible to define the second normal stress difference as:

(5) $$N_{2}=\tau_{yy}-\tau_{zz},$$

where $\tau_{zz}$ is the normal component of the stress tensor along the vorticity direction. For diluted and semidiluted polymer solutions, the second normal stress difference displays lower values than the first normal stress difference [2], and it is very challenging to measure [9].

Before concluding this section, it is also important to report some basic principles of capillary rheometry, as many microfluidic devices take advantage of the channel configuration similar to the capillary rheometer. Capillary rheometers are basically straight capillaries with radius *R* and length *L*, where the fluid flows subjected to a pressure drop ΔP (Figure 1f). In these conditions, it is possible to define the wall shear stress as [2]:

(6) $$\tau_{w}=\frac{\Delta P}{L}\frac{R}{2},$$

while the wall shear rate for a Newtonian liquid is defined as:

(7) $${\dot{\gamma }}_{aw}=\frac{4Q}{\pi R^{3}},$$

where *Q* is the volumetric flow rate. The viscosity for a Newtonian liquid is obtained as $\eta =\tau_{w}/{\dot{\gamma }}_{aw}$, which is also the Hagen–Poiseuille equation [2,7]. For non-Newtonian fluids, the situation is not straightforward, as the velocity profile in the channel depends upon the flow-rate and, consequently, upon the shear rate. In these cases, the Weissenberg–Rabinowitsch equation [2] can be used to evaluate the wall shear rate ${\dot{\gamma }}_{w}$:

(8) $${\dot{\gamma }}_{w}={\dot{\gamma }}_{aw}\left(3+\frac{d\ln Q}{d\ln\tau_{w}}\right).$$

## 3. Microfluidic Rheometry in Shear Flow

There is a plethora of microfluidic devices introduced to measure rheological properties in shear flow, especially those to quantify the shear viscosity. Over the years, different types of approaches were introduced based on new developments in the understanding of physics phenomena at the microscales, as well as on technological advancements in the field of sensing or 3D printing. In this section, microfluidic rheometers are divided according to their working principle, starting from those that include sensing components, to those based on particle tracking. The interested reader may also look at previous reviews on the subject [10,11].

### 3.1. Micro-Electro-Mechanical Systems (MEMS)

**Figure 2:**
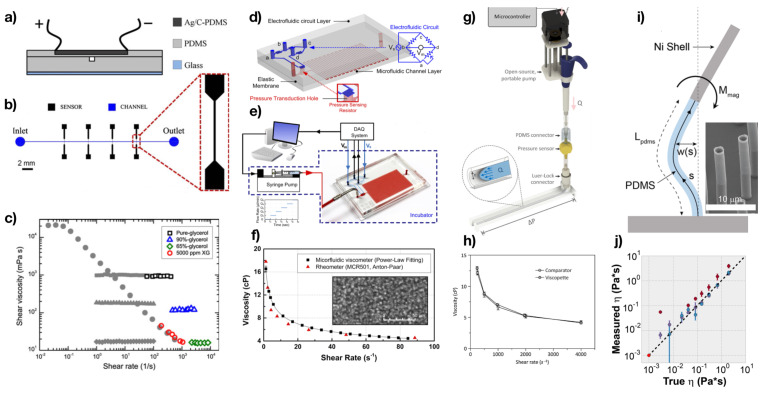
Examples of Micro-Electro-Mechanical Systems (MEMS) employed in microfluidic rheometry. (**a**,**b**) Schematic of the microfluidic rheometer made of PDMS with pressure sensors also made of flexible PDMS containing silver and black carbon conductive particles. (**c**) Good agreement between conventional and microfluidic rheometry data using the apparatus in (**a**,**b**). Reprinted with permission from Springer Nature: Rheologica Acta, Pan and Arratia, Copyright (2013) [17]. (**d**,**e**) Schematic of the microfluidic device with an electrofluidic circuit employed as pressure sensor. (**f**) Good agreement was observed between bulk and microfluidic viscosity data for whole blood samples. The inset is a real-time image of blood cells in the microfluidic channel. Reprinted with permission from Lee et al., Analytical chemistry, 90, 2317–2325 [18]. Copyright 2018, American Chemical Society. (**g**) Schematic of the hand-held, automatic capillary viscometer. (**h**) Good agreement was observed between bulk and microfluidic viscosity data for xanthan gum 1.0 wt% solutions. Reprinted from Sensor and Actuators:B, 313, 112176, Lee et al., hand-held, automatic capillary viscometer for analysis of Newtonian and non-Newtonian fluids [19], Copyright (2020), with permission from Elsevier. (**i**) Schematic of a microfluidic viscometer using magnetically actuated micro post arrays. (**j**) Good agreement was observed between bulk and microfluidic viscosity data for sucrose and Karo solutions. Reprinted from Judith et al. [20].

Micro Electro Mechanical Systems, hereafter MEMS, are small systems containing electromechanical components with sizes on the order of 100s of microns and below [12]. MEMS systems have found large application in microfluidic rheometry, in light of their compact sizes and sensitivities. Kang et al. [13] presented a microfluidic slit rheometer with a pressure sensor to measure the viscosity curve of polymer solutions at shear rate values up to $\dot{\gamma }\approx 10^{4}\;s^{-1}$. They found good agreement between conventional and microfluidic data for polyethylene oxide solutions and hydroxyethyl cellulose solutions. The slit rheometer was made of glass and it presented several pressure sensors embedded on the bottom wall of the microfluidic device. This system was based on the well-established capillary rheometer previously derived for polymer melts [2,14]. Pipe et al. [15] compared rheological measurements performed using a conventional rheometer with those derived using a microfluidic slit rheometer produced by Rheosense. By using a syringe pump to control the volumetric flow rate, the pressure at the wall as a function of the distance from the device inlet could be monitored thanks to a pressure sensor mounted on the device wall, thus then leading to the measurement of the shear viscosity at different values of the shear rate. The authors found very good agreement between the data obtained using conventional rheometry and those obtained via the microfluidic rheometer. They also observed, as expected, that the microfluidic rheometer was able to reach imposed values of the shear rate up to $\dot{\gamma }\approx 10^{4}\;s^{-1}$, an order of magnitude above the values explored by conventional rheometers. Another system was recently developed by Maurya et al. [16], where the fluid flowed without the need of a syringe pump in a microfluidic chamber made of a top part of glass and a bottom part of oxidised silicon wafer. This device was used to measure a single value of viscosity for several diesel/biodiesel compositions, and the resulting data were compared with those available from the literature, finding good agreement, as show in Figure 2.

Very recently, Puneeth et al. [21] employed 3D printing to fabricate a microfluidic rheometer with integrated electromechanical parts. This device was used to measure a single value of the viscosity for samples containing lysozyme, human serum albumin and bovine serum albumin, all having a viscosity falling in the range of 0.5–10 cP. Based on the data presented so far, both the microfluidic rheometer introduced by Maurya et al. [16] and by Puneeth et al. [21] are currently usable for rapid order-of-magnitude measurements rather than detailed rheological characterisation. Pan and Arratia [17] presented a microfluidic slit rheometer made of polydimethylsiloxane (PDMS), where the pressure sensor on the upper wall was also made of a flexible PDMS membrane containing silver and black carbon particles (Figure 2a,b). The advantage of this microfluidic rheometer over the one made of glass was the fact that it could be fabricated using standard lithography tools [22]. Owing the flexible nature of the membrane, it was deformed during the flow of the liquid inside the channel, providing a measure of the pressure at the wall. The authors found good agreement between conventional and microfluidic rheometry data for both Newtonian and non-Newtonian fluids (Figure 2c), with the microfluidic rheometer reaching shear rate values up to $\dot{\gamma }\approx 10^{4}\;s^{-1}$. A similar device featuring deformable flexible membranes was introduced by Liu et al. [23] for the measurement of a single value of the viscosity for mineral oil, blood and water, while also comparing single-values of blood viscosity for anaemia, normal, and polycythemias bovine blood. A variation to this system was introduced by Lee et al. [18]. The authors here employed an electrofluidic circuit with conductive resistors containing ionic liquids as a pressure sensor (Figure 2d,e). The advantage of this approach over previous ones was the fact that ionic liquids provided better thermal stability, an important requirement when attempting to perform rheological measurements at different temperatures. During flow, the walls of the microfluidic devices where bent because of the wall stress, causing a change in the electrical resistance of the resistor. After performing an accurate calibration, the authors employed their microfluidic rheometer to measure the viscosity values of Newtonian glycerol solutions, as well as non-Newtonian solutions such as whole blood at different temperatures (25 ${}^{{}^{\circ}}$C and 37 ${}^{{}^{\circ}}$C), finding good agreement with conventional bulk measurements (Figure 2f). A similar system was also introduced by Tzeng and Sun [24] for the measurement of glycerol-water solutions at different volume concentrations. A portable microfluidic rheometer called Viscopette was introduced by Lee et al. [19] (Figure 2g). The setup was very simple, and it required an open-source pump, an inline pressure sensor and a cut Tygon tube. The authors employed the Viscopette to measure the viscosity of Newtonian and non-Newtonian fluids at different shear rate values, finding good agreement with conventional rheometry (Figure 2h). A different type of microfluidic rheometer made of very easy laboratory tools was introduced by Hudson et al. [25]. The authors employed their capillary rheometer for the measurement of the viscosity of protein solutions at different concentrations and temperature, finding good agreement with conventional bulk measurements. A conceptually different microfluidic rheometer was developed by Judith et al. [20], who employed magnetically actuated micropost arrays to measure the fluid viscosity in the absence of flow (Figure 2i). The posts were made of a PDMS base (to allow flexibility) with an apex of nickel (Ni). When applying a magnetic field, the Ni-based post would deflect by different amounts depending on the magnetic field applied. By using a numerical model developed by the authors, they linked the post deflection to the fluid viscosity, demonstrating good agreement with conventional bulk techniques for sucrose and Karo solutions. A similar device was also reported by Mustafa et al. [26], who made the pillars fully flexible and tracked their deflection during the flow inside the microchannel and linked the deflection to the viscosity of the flowing liquid. They applied their device to the viscosity measurement of decanol, aqueous glycerol solutions and whole blood.

### 3.2. Interface-Based Microrheometry

**Figure 3:**
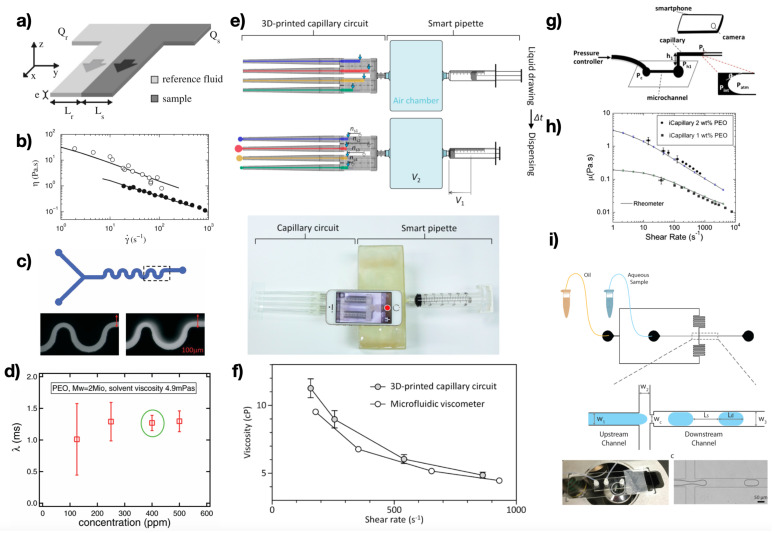
Examples of microfluidic rheometers based on interfacial phenomena. (**a**) The interface between a reference Newtonian liquid and a sample is studied to obtain the viscosity of the sample liquid. (**b**) Measurements carried out using the device in (**a**) on PEO solutions at 2 and 4 wt%. Reprinted with permission from Springer Nature, Microfluidics and Nanofluidics, Guillot and Colin, Copyright (2014) [27]. (**c**) Serpentine microfluidic device for the measurement of longest relaxation time in curved microfluidic devices. (**d**) Measurement of longest relaxation time for several PEO solutions in the dilute regime. Reprinted from Zilz et al. [28]. (**e**) Schematic of the 3D printed capillary circuit microfluidic rheometer. (**f**) Good agreement was found between the 3D printed capillary circuit data and those obtained using another microfluidic rheometer. Reprinted from Oh et al. [29]. (**g**) Schematic of the iCapillary device based on monitoring the sample air interface using a smartphone. (**h**) Good agreement was found between iCapillary data and conventional rheology data for PEO solutions. Reprinted with permission from Springer Nature, Rheologica Acta, Solomon et al., Copyright (2016) [30]. (**i**) Microfluidic rheometer based on the droplet formation mechanisms in flow-focusing geometry. Reprinted with permission from Li et al., Analytical Chemistry, 89, 3996–4006 [31]. Copyright 2017, American Chemical Society.

As suggested by the term, interface-based microrheometers are those where an interface is established. These are divided in coflow devices, where two miscible liquids flow side by side; air–liquid tracking devices, where the interface between the sample and a gas (generally air) is tracked over time; and the droplet-based systems, where two nonmiscible liquids form droplets at a junction, and this droplet is tracked over time. These categories of devices are now reviewed in separate paragraphs, as shown in Figure 3.

#### 3.2.1. Coflow

A first category of devices falling in this group is the one where the shear viscosity is measured by monitoring the interface among two liquids, the sample liquid and a reference fluid. Choi and Park [32] introduced a microfluidic device based on this principle to measure the viscosity of protein solutions at different concentrations and temperatures. Guillot and Colin [27] studied the interface between a reference Newtonian fluid and several solutions of complex liquids (Figure 3a,b). The position of the interface depended upon the ratio between the two volumetric flow rate values, of the reference and of the sample liquid; this information was used to derive the pressure gradient and the wall shear-rate. By further introducing the ‘Weissenberg Rabinowitsch Mooney method’ [2], they measured the viscosity for PEO solutions at 2 and 4 wt% (Figure 3b), micellar solutions and Brij 30 solutions. The advantage of their technique over MEMS is the fact that no sensor is required; a disadvantage, however, is the fact that multiple channels together with a reference liquid are required. The device introduced by Guillot and Colin was similar to the one previously introduced by Nguyen et al. [33], where the main difference being the fact that the device by Nguyen et al. was based on ‘sandwiching’ the sample liquid between two reference sheath fluids. The authors performed measurements of PEO solutions to prove the applicability of their device. The angle with which the two fluids ‘met’ at the junction had an important impact on the measurement accuracy, as reported by the numerical simulations of Kang et al. [34]. Several follow-up studies followed these original works to measure the viscosity of fluids and biofluids. Kim et al. [35] replaced the use of a conventional camera with a smartphone camera and performed viscosity measurements on blood samples and several samples containing difference oil/rancid oil amounts. Hong et al. [36] employed 3D-printing fabrication techniques to fabricate a coflow microfluidic rheometer and measured the viscosity values for glycerol–water solutions, as well as blood samples from healthy volunteers and from diabetic patients. Kang [37] introduced a coflow system to measure blood viscosity and red blood cell aggregation in vitro using a closed-loop circulation system. A similar system was also introduced to measure blood viscoelasticity [38]. Hinternuller et al. [39] attempted to remove the requirement of a camera to track the interface between the two fluids by employing a capacitive sensor embedded in the microfluidic device, similarly to the MEMS devices discussed before. They demonstrated the validity of their device by deriving the viscosity of several water/glycerol mixtures. Kang and Yang [40] introduced a coflow device where a temperature controller was also embedded in the microfluidic rheometer to measure the viscosity of blood in plasma and phosphate buffer saline. The microfluidic rheometers presented so far displayed a major limitation of measuring only the shear viscosity. Zilz et al. [28] were among the firsts to introduce a microfluidic device for the measurement of the longest relaxation time of dilute PEO solutions. Their microfluidic device presented a serpentine geometry aimed at generating a viscoelastic instability [41] at the interface between two identical viscoelastic liquids coming from two different streams (Figure 3c). Above a critical value of the imposed flow rate, the two fluids displayed an elastic instability that could be modelled according to the Pakdel–McKinley criterion [42]. Zilz et al. [28] demonstrated the measurement of the longest relaxation time for several PEO solutions in the dilute regime. The importance of the device introduced by Zilz et al. is the fact that the longest relaxation time for diluted and semidiluted unentangled polymer solutions cannot generally be measured using conventional bulk techniques because of technological limitations [3].

#### 3.2.2. Air–Liquid Interface Tracking

In addition to the coflow geometries featuring a reference fluid and the sample, other microfluidic rheometers allowed viscosity measurements via the tracking of an air–liquid interface. Oh and Choi [29] introduced a 3D-printed capillary circuit (Figure 3e,f) to measure the shear viscosity of several glycerol (Newtonian) and xanthan gum (non-Newtonian) aqueous solutions. While flowing in the capillary circuit, liquid displaced the air initially contained in the capillaries; the flow rate could be measured by tracking the fluid–air interface thanks to a small scale. The pressure drop was instead measured by applying Boyle’s law for ideal gas to the air chamber. The authors found good agreement between their xanthan gum measurements using their 3D-printed capillary circuit and another microfluidic viscometer. A similar principle was also used by Phu-Pham et al. [43] to measure the viscosity of several liquids, including glycerin–water solutions, acetone and milk, and by Han et al. [44] to measure the viscosity of several Newtonian liquids. Zou et al. [45] employed microwire molding to fabricate a microfluidic rheometer made of PDMS. The resulting device was introduced in a water bath to keep the temperature constant, while the air–liquid interface was tracked using a camera. The volumetric flow rate could be derived by measuring the liquid displacement over time, while the pressure was evaluated using a sensor. The authors employed this device to measure a single viscosity value for several Newtonian solutions. Solomon et al. [30] introduced the iCapillary, a microfluidic rheometer based on the tracking of the interface between the sample and the liquid using a smartphone camera (Figure 3g). The authors successfully employed this device to measure the viscosity of PEO solutions at 1 and 2 wt% (Figure 3h), finding good agreement between their data and the conventional rheometry data. They also measured several bovine serum albumin solutions at different concentrations and found good agreement with the literature data. In the attempt of removing the dependency from optical cameras, Mendez-Mora et al. [46] embedded an electronic sensor to detect the liquid–air front. They employed their device to measure the shear viscosity of blood at different hematocrits levels across two order of magnitude in shear-rate. Very recently, Tammaro et al. [47] introduced a microfluidic capillary for the simultaneous measurement of shear viscosity and first normal stress difference as a function of the imposed shear rate. The authors tracked the flow of polymer melts exciting from the microchannel using a camera equipped with an objective. The same device could be used to quantify several other phenomena and parameters, including extrudate swell, contact angle and melt fracture. The work by Tammaro and coworkers marked a departure from the use of standard PEO solutions for microfluidic rheometry testing, while opening to a new area of microfluidic rheometry in the context of polymer melts, something that was not addressed before. The further possibility of performing simultaneous measurements of both viscous and elastic properties using few milligrams of sample makes microfluidic rheometry appealing compared to the well-established bulk rheometry.

#### 3.2.3. Droplet-Based Systems

Other types of microfluidic rheometers have been introduced with a working principle based on the droplet formation mechanism when two nonmiscible fluids meet at a junction. DeLaMarre et al. [48] employed a T-junction microfluidic device and demonstrated viscosity measurements in the range 0.96–52 cP for the dispersed aqueous phase. Their technique was based on the simple measurement of the aqueous droplet length and of the spacing between two consecutive aqueous droplets. More recently, a similar microfluidic setup was used to measure the viscosity of silicone oil for different T-junction geometries [49]. A T-junction microfluidic device was also used to evaluate the change in viscosity due to the growing of a bacterial colony in a microdroplet [50]. A device based on the same working principle but involving a flow-focusing geometry was introduced by Li et al. [31] (Figure 3i). The authors demonstrated viscosity measurements for both Newtonian and non-Newtonian solutions, finding good agreement among their measurements and the conventional rheology data. A similar device was used by Deng et al. [51] in the context of oil quality for the food industry. Recently, Mena et al. [52] employed a flow-focusing microfluidic device to measure blood coagulation over several minutes, by employing blood (sample) and oil (continuous phase). Coagulation was quantified by monitoring shear viscosity changes over several minutes.

### 3.3. Particle Tracking Microrheometry

**Figure 4:**
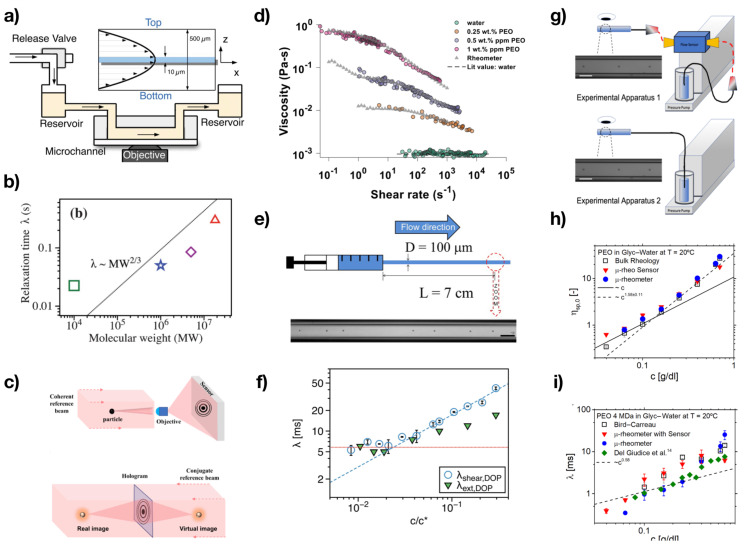
Examples of microfluidic rheometers based on particle tracking. (**a**) Schematic of a microfluidic device for creep measurements, aimed at deriving the longest relaxation time λ. (**b**) Measurements of λ as a function of the polymer molecular weight for several polyacrylamide solutions, using the microfluidic rheometer in (**a**). Reprinted from Reference [56], with permission of The Royal Society of Chemistry. (**c**) Schematic of the microfluidic rheometer based on digital holography microscopy. (**d**) Good agreement between microfluidic and conventional rheometry data for several PEO solutions. Reprinted with permission from Gupta and Vanapalli [58], with the permission of AIP publishing. (**e**) Schematic of the μ-Rheometer microfluidic rheometer, based on the transversal migration of rigid particles flowing in microchannels. (**f**) Good agreement between the μ-rheometer and the OSCER [69] was found for polystyrene solution in dioctyl phthalate. Reprinted from Del Giudice et al. [64]. (**g**) Schematic of the μ-rheometer for the simultaneous measurement of zero-shear viscosity and longest relaxation time. (**h**) Good agreement was observed between microfluidic and bulk zero-shear viscosity data for several PEO solutions. (**i**) Good agreement was observed between microfluidic and bulk longest relaxation time data for several PEO solutions. Reprinted from Del Giudice [68].

Particle tracking has often been used to evaluate the viscoelastic properties of a fluid by monitoring the Brownian motion of particles suspended in the stationary fluid under investigation. Such techniques mainly fall under the category of ‘microrheology’ [53,54] rather than microfluidic rheometry, thus not being included in this review. However, there are several microfluidic rheometers that have been introduced to evaluate rheological properties by direct tracking of particles under imposed flow, and these fell within the remit of this review. Drost and Westerweel [55] developed a microfluidic rheometer for the evaluation of the flow index of xanthan gum solutions by visualising the streamlines in an microfluidic device with expansion geometry. Koser et al. [56] introduced a microfluidic rheometer to measure the longest relaxation time of polyacrylamide solutions at different molecular weights (Figure 4a,b). The microfluidic rheometer featured a simple straight channel where the flow was imposed via a pressure pump connected to a release valve. The velocity profile in the channel was Poiseuille-like, and it was experimentally derived by tracking fluorescent 3μm particles, which were not affecting the local flow. While flowing, the liquid sample containing the particles was subjected to a value of external stress depending upon the value of the imposed pressure drop; when the release valve was opened, the fluid begun to relax the stress and, by tracking the resulting motion of the fluorescent particles, the authors derived a strain curve for the material. From this curve, they estimated the longest relaxation time of all their solutions, finding good agreement with the theoretical predictions from the finite extensibility nonlinear elastic (FENE) constitutive equation. Another microfluidic rheometer was introduced by Vishwanathan and Juarez [57] to measure the kinematic viscosity of Newtonian solutions. The working principle of this work was based on subkilohertz liquid oscillations near cylindrical obstacles. By tracking the motion of microparticles in the proximity of the cylinders, the authors measured the kinematic viscosity of a 30 wt% glycerol–water solution, acetone and ethanol. A microfluidic rheometer based on digital holography microscopy was recently introduced by Gupta and Vanapalli [58] (Figure 4c,d). They employed particle tracking with holographic particle position reconstruction to derive the viscosity curve of several PEO solutions using less than 10μL of sample and finding excellent agreement with conventional rheology data (Figure 4d). Del Giudice et al. [59] introduced a microfluidic rheometer for the measurement of the longest relaxation time (Figure 4e), based on the well-known phenomenon of transversal migration of particles in straight microchannels [60,61]. The flow was controlled using a syringe pump, and the relaxation time was evaluated from the fraction of particles aligned on the channel centreline (experimental snapshot in Figure 4e) thanks to the theoretical model introduced by Romeo et al. [62]. This microfluidic rheometer, also called μ-rheometer, was employed for a variety of liquids, including PEO and polyacrylamide aqueous solutions [59,63], hyaluronic acid and chitosan aqueous solutions [64,65], polystyrene solutions in good (tricresyl phosphate) and theta solvents (dioctyl phthalate, Figure 4f) [64], aqueous solutions of hydroxyethyl cellulose [66] and polymerised ionic liquids in ionic liquid solutions [67]. The main limitation of the μ-rheometer was the fact that only the longest relaxation time could be measured. Very recently, Del Giudice [68] demonstrated the first microfluidic rheometer for the simultaneous measurement of zero-shear viscosity and longest relaxation time (Figure 4g). The working principle was the same as the original μ-rheometer; however, here, the syringe pump was replaced by a pressure pump. Since the pressure drop was now known and controlled via the pressure pump and the flow rate was evaluated via particle tracking, the viscosity could be measured using the Hagen–Poiseuille equation, while the longest relaxation time was evaluated following the original procedure for the μ-rheometer. Good agreement was observed between microfluidic and bulk rheology data for several PEO solutions (Figure 4h,i).

### 3.4. Paper-Based Microfluidic Rheometers

Paper microfluidics has also contributed to the development of microfluidic viscometers. Some advantages of paper microfluidics over conventional techniques include their relatively inexpensiveness and their suitability for applications in analytical chemistry [70,71]. Kang et al. [72] introduced a paper microfluidic rheometer based on the coflow between a reference fluid and the sample. The viscosity measurement was based on the change in colour of the paper strip. They successfully employed their device for the measurement of several glycerol–water solutions, as well as biofluids including saliva, blood plasma and bovine serum albumin. The accuracy of paper-based microfluidic rheometers have been also reported in the literature for devices made of cellulose [73]. Traditional paper-based microfluidic devices can only process relatively small flow rate values due to their working principle. Jang et al. [74] employed fast-flow paper based devices made of two layers of paper rather than one, thus reducing the measurement time. They employed their microfluidic device to measure the viscosity of polyethylene glycol aqueous solutions at different concentrations and of artificial saliva. New directions in paper-based microfluidic are aimed at employing 3D-printing technologies [75]. For instance, Puneeth and coworkers [21,76] recently employed 3D printing to fabricate a paper-based microfluidic viscometer for the measurement of biofluids, including saliva and bovine serum albumin.

## 4. Microfluidic Rheometry in Extensional Flow

As described previously, the extensional flow is significantly different from the shear flow, meaning that the response of the material to the extensional flow can be different compared to the shear flow [2]. The field of extensional rheometry is very vast, and here, only some microfluidic devices employed for the rheological characterisation of solutions are highlighted; the interested reader can also look at the detailed review by Haward [77] and at the references therein.

**Figure 5:**
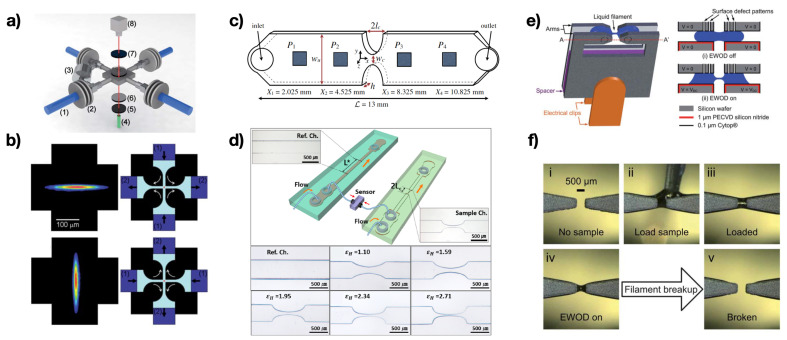
Examples of extensional microfluidic rheometers. (**a**) Schematic of the cross-slot microfluidic device, with two inlets and two outlets. (**b**) Flow birefringence measurements of polystyrene in dioctyl phthalate solutions. Different flow conditions are represented. Reprinted with permission from Reference [69], with permission of The Royal Society of Chemistry. (**c**) Schematic of the hyperbolic-contraction extensional rheometer. Reprinted with permission from Springer Nature, Rheologica Acta, Ober et al., Copyright (2013) [91]. (**d**) Schematic of the differential pressure extensional rheometer. The experimental snapshots represent different types of hyperbolic contractions channel compared to the straight reference channel. Reprinted with permission from Kim et al., Copyright (2018), The Society of Rheology [92]. (**e**) Schematic of the miniature capillary breakup extensional rheometer, where the liquid bridge is generated using electrowetting-on-dielectric actuation. (**f**) Experimental snapshots of the loading step for the device in (**e**). Reprinted with permission from Reference [93], with permission of The Royal Society of Chemistry.

One of the first microfluidic devices to study the extensional flow properties of complex fluids was the cross-slot [78] (Figure 5a,b). The device featured two inlets and two outlets (Figure 5a), with the flow controlled by independent pressure pumps. The extensional flow was obtained in the central part of the channel where the middle point was a stagnation point. A birefringent setup was then employed to visualise the birefringent signal from the flowing solution. The birefringent signal depends upon the flow orientation, and it could be displayed either horizontally or vertically (Figure 5b). The cross-slot was widely used to characterise the extensional properties of several complex fluids, including polystyrene solutions [69], saliva [79] and polyacrylamide solutions [80]. The cross-slot geometry was very successful in measuring extensional properties, and, for this reason, several studies attempted to identify the optimal shape of the middle area of the microfluidic device in order to achieve a ‘pure’ extensional flow [81,82]. For instance, the Optimised Cross-Slot Extensional Rheometer (OSCER) was introduced by Haward et al. [81], and it was further used to characterise hyaluronic acid solutions [83] and polystyrene solutions [64]. Cross-slot geometries were also used to generate viscoelastic droplets and to analyse the filament stretching experienced by viscoelastic droplets formed in flow-focusing geometries. For instance, Juarez and Arratia [84] employed the cross-slot as a flow-focusing droplet microfluidic device to evaluate the extensional properties of λ-DNA at different molecular weights, being able to quantify the extensional viscosity of the solutions. More recently, Marshall and Walker [85] employed a microfluidic T-junction device for the generation of viscoelastic droplets containing the sample, followed by a cross-slot device where the droplet was vertically stretched, leading to an extensional flow. They employed their device to characterise several Newtonian (glycerol–water) and non-Newtonian (PEO) aqueous solutions. The cross-slot geometry was also used together with the ‘Stokes trap’ principle [86] to study the large amplitude oscillatory shear (LAOS) of single DNA molecules [87]. This same device [87] was also used together with passive microrheology principles [88,89] to derive the extensional viscosity of PEO solutions and λ-DNA solutions. Very recently, a combination of extensional and shear microfluidic devices was used to study the alignment of colloidal rods [90].

Another way of generating an extensional flow in microfluidic devices is by using an hyperbolic-contraction device (Figure 5c). In such devices, pressure sensors are located before and after the contraction; different pressure readings are related to the extensional properties of the fluid, thus providing an accurate measurement, while also allowing optical access via conventional microscopy [91]. This device was used to characterise the extensional flow of PEO solutions [94], surfactant solutions [95], and methyl cellulose solutions [96]. Tiny variations of the hyperbolic-contraction channel featuring a longer middle section where the fluid could be stretched further were also introduced to study extensional properties of PEO and polyacrylamide solutions [97], as well as bioparticles and actin filaments [98]. A slightly different concept featuring the comparison between the flow in a straight channel and another in the hyperbolic-contraction geometry was also introduced by Kim and coworkers [92]. This device was called differential pressure extensional rheometer, and it was based on the comparison of the pressure difference between the converging channel and the reference straight channel (Figure 5d). The authors employed this device to first characterise the extensional viscosity [35] and then the longest relaxation time [99] of several PEO solutions in diluted and semidiluted conditions.

Before concluding this section, it is worth mentioning the microfluidic capillary breakup extensional rheometer (CaBER) introduced by Nelson et al. [93]. The device was a miniaturisation of the conventional CaBER instrument, where the liquid bridge responsible for the filament formation was formed thanks to electrowetting-on-dielectric forces, rather than mechanical plate separation (Figure 5e,f). The sample was first loaded between the patterned surfaces (Figure 5e,f); when the electrowetting-on-dielectric force was activated, the filament stretching begins, thus leading to a similar dynamics as for the CaBER. The authors employed this device to characterise several glycerol and PEO aqueous solutions.

## 5. Microfluidic Rheometry Integrated to SANS and SAXS

Microfluidic rheometry presents, among others, two advantages compared to conventional bulk techniques: the easy optical access and the easy integration with other technologies. Such advantages have been widely exploited in the context of Small Angle Neutron Scattering (SANS) and Small Angle X-ray Scattering (SAXS). Such integration led to the rheological characterisation of the sample together with detailed microstructure information, which is difficult, or even impossible, to obtain using conventional rheometry. Here, some of these devices are reviewed, but the interested reader can find additional information in the perspective article by Silva [100], the reviews by Ghazal et al. [101] and Bharati et al. [102], together with the references therein.

**Figure 6:**
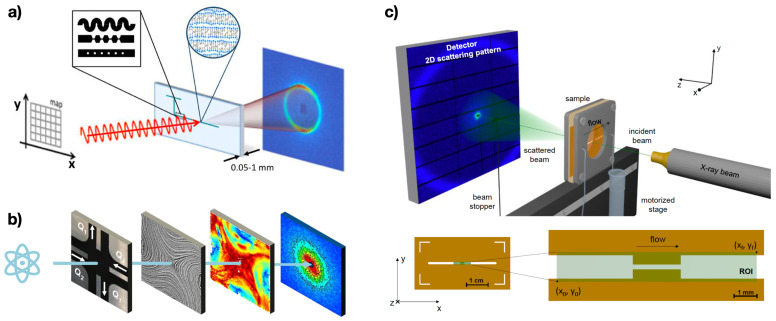
Examples of microfluidic rheometers integrated to Small Angle Neutron Scattering (SANS) and Small Angle X-ray Scattering (SAXS). (**a**) Schematic of SANS integrated to a microfluidic device. Reprinted from Reference [103]. (**b**) Schematic of SANS experiments performed on a four-mill microfluidic device. Reprinted from Reference [110]. (**c**) Schematic of a SAXS apparatus integrated to a microfluidic device. Reprinted with permission from Reference [111], with permission of The Royal Society of Chemistry.

SANS experiments were first integrated to microfluidic devices by Lopez et al. [103]. The laser beam was oriented towards the microfluidic device, and the resulting spectrum was subsequently analysed (Figure 6a). The authors performed experiments on two model complex fluids, namely cetyl trimethylammonium chloride/pentanol/D${}_{2}$O and sodium lauryl sulfate/octanol/brine lamellar systems. In addition to studying the orientation dynamic of these systems in contraction-expansion geometries, they also provided an ‘application diagram’ for SANS microfluidic relating acquisition time and beam intensity for different applications. The performance of the SANS microfluidic apparatus was also found to depend upon the material of the microfluidic device and, consequently, of the fabrication method [104]. SANS microfluidic systems were also used to study the orientation of worm-like micellar solutions in both a simple straight slit-channel [105] and in a contraction-slit geometry [106]. SANS was also coupled to capillary rheometer in a setup called ‘Capillary RheoSANS’ [107]. This arrangement took advantage of the SANS investigation together with the high shear rate values generally achievable in capillary microfluidic rheometers. The authors employed this system to study the chaining of silica nanoparticles, the alignment of wormlike micelles and the aggregation of monoclonal antibodies. By coupling this technique to the conventional RheoSANS apparatus, they demonstrated the possibility of studying the material microstructure over 8 orders of magnitude in the imposed shear-rate. Very recently, microfluidic SANS was used to study the lamellar-to-multilamellar transformation in a model surfactant system made of sodium dodecyl sulfate, octanol and brine [108]. SANS is often affected by the problem of sequential measurements, where long time is required to move from one sample to the next. This problem was partially addressed by Adamo and coworkers [109], who designed a microfluidic mixer where several samples could be analysed without the need of disassembling the whole apparatus. This approach remains very useful for the microstructure analysis of complex fluids at different concentrations.

SANS was also employed in conjunction with flexible microfluidic devices to study complex fluids (Figure 6b) [110]. Corona and coworkers [110] employed a microfluidic version of the macroscopic four-roll mill, where different flow conditions, including shear-flow, extensional flow and rotation flow, could be imposed by simply changing the flow directions [112]. The addition of SANS allowed the authors to study the flow-induced orientation of cellulose nanocrystal dispersions under a wide range of 2D deformations.

Microfluidic devices were also integrated with SAXS apparatus, similarly to the SANS, to study the flow behaviour of several complex liquids (Figure 6c). The first experimental integration of SAXS and microfluidic was presented by Trebbin and coworkers [113], who employed a microfluidic contraction geometry to study the alignment of cylindrical micelles. Several other works have employed contraction geometries for similar studies. Poulos et al. [114], for instance, employed this configuration to study linear sodium alkylbenzenesulfonate (NaLas) surfactant. They experimentally showed the heterogeneous character of the NaLas solutions, especially when flowing inside a contraction. Buscema and coworkers [115] employed a contraction device to explore the flow-induced structural changes of liposomes. Very recently, Rodriguez-Palomo et al. [116] employed SAXS to study the structure of lyotropic liquid crystals and their behaviour in contraction microfluidic devices. Few other studies were also reported to explore the flow behaviour of complex fluids in geometries substantially different from the contraction one, including serpentine channels and microfluidic pillars [111,117]. Before concluding this section, it is also worth reporting that a complementary use of SANS and SAXS was also used to provide more details regarding the flow of micellar solutions [118].

## 6. Conclusions and Perspectives

An overview of the microfluidic devices reviewed here together with their working principles is reported in Table 1. The advantages of microfluidic devices over conventional macroscopic techniques, including little sample required, easy integration and optical access, made them appealing for the rheological characterisation of complex fluids. Despite that, microfluidic rheometry is somehow at its infancy, with only a few devices that were either exploited commercially or in advanced research applications. In terms of microrheometers in shear flow, the m-VROC produced by Rheosense [15] remains the main microfluidic device that has both been commercialised and it has also been used for research in the rheology field. One disadvantage of the m-VROC is the fact that only the shear viscosity can be measured. The μ-rheometer introduced by the author of this review [68] allowed the simultaneous measurement of zero-shear viscosity and longest relaxation time; however, even though it has been employed on a wide portfolio of samples, it has not been widely applied across different research groups (nor commercialised). Moreover, the μ-rheometer cannot be used to characterise shear-thinning liquids, Newtonian liquids and suspensions. In some ways, the μ-rheometer is an instrument that can be used in addition to conventional rheometers to evaluate unentangled polymer solutions or biofluids properties, in the limit where conventional rheometry tends to fail because of technical limitations. Other microfluidic rheometers for the measurement of the longest relaxation time in shear flow, such as those introduced by Zilz et al. [28] and Koser et al. [56], have not been employed aside from their original proof-of concept, and future investigations on other polymer solutions are still required. Other microfluidic rheometers in shear flow have instead been introduced with the aim of studying biological fluids but without the ambition of framing the results within the rheological framework at large. Such approach makes them not ready for use in advanced rheological studies, such as macromolecule conformation in solutions. A problem that could be addressed using microfluidic technologies is the measurement of the second normal stress difference $N_{2}$, which remains challenging [9]. One approach could be based on the study of the particle migration patterns in straight channels, as this has been demonstrated to change as consequence of secondary flows in microfluidic devices [119,120,121]. So far, however, it has not been possible to infer $N_{2}$ from the particle migration pattern, thus leaving this problem open to new solutions.

While microfluidic rheometers in shear flow still require improvements, extensional microrheometers have been widely used for the analysis of single molecules, as well as for the study of bioparticles. In this case as well, Rheosense commercialised the e-VROC, which is the extensional version of the m-VROC for shear flow. The rheological community has been very engaged in developing extensional microfluidic rheometers, thus leading to a portfolio of devices whose results were strongly linked to the well-established polymer physics framework [8]. Similarly, the combination of SANS and SAXS with microfluidic rheometer led to the simultaneous analysis of microstructure evolution under confined flow together with rheological analysis.

Future efforts should also look into machine learning and artificial intelligence, which has recently been the focus within the microfluidic community at large [122]. Integration of such novel methods into existing or new microrheometers can lead to the development of efficient platforms for detailed rheological study, as well as for rapid, order-of-magnitude, estimations of rheological parameters, useful in the medical or industrial practice.

## Figures and Tables

**Table 1:** Overview of the microfluidic rheometers reviewed here.

Working Principle	Class	Flow Type	Pros	Cons	References
Slit/capillary rheometry	MEMS	Shear	Large shear rate values. Closed system.	Complex fabrication.	[13,15,16,17,18,19,21,23,24,25]
Deflection of pillars under flow	MEMS	Shear	Easy to replace.	Complex calibration.	[20,26]
Coflow	Interface based	Shear	Clear working mechanism.	Reference fluid required.	[27,28,32,33,35,36,37,38,39,40]
Air–liquid interface tracking	Interface based	Shear	Easy setup.	Modelling required.	[29,30,43,44,45,46,47]
Droplet based	Interface based	Shear	In-drop phenomena evaluation.	Second liquid required. Droplet instabilities.	[31,48,49,50,51,52]
Particle tracking	Interface based	Shear	Measurement of η and λ.	Particle addition required. Complex setup.	[55,56,57,58,59,63,64,65,66,68]
Paper-based devices	Interface based	Shear	Simple setup	Multiple devices for multiple measurements.	[21,72,73,74,75,76]
Cross-slot extension	Birefringence	Extensional	Single polymer chain analysis.	Birefringence required.	[64,69,78,79,80,81,82,83]
Cross-slot droplet formation	Droplet formation	Extensional	Evaluation of in-drop phenomena.	Second liquid required. Droplet instabilities.	[84,85]
Cross-slot with Stokes trap	Microrheology	Extensional	Extensional viscosity measurement.	Complex setup and particle tracking.	[86,87]
Hyperbolic contraction flow	MEMS	Extensional	Bioparticle and single filament analysis.	Complex fabrication.	[91,94,95,96,97,98]
Hyperbolic contraction comparative flow	Differential pressure	Extensional	λ measurement	Multiple devices required.	[35,92,99]
CaBER	Electrowetting on dielectric force	Extensional	Capillary breakup measurements.	Complex setup.	[93]
SANS integration	Laser beam	Shear and Extensional	Microstructure characterisation.	Complex setup.	[103,105,106,107,108,109,110,112]
SAXS integration	Laser beam	Shear and Extensional	Microstructure characterisation.	Complex setup.	[111,113,114,115,116,117,118,118]
